# Racial differences in length of stay and readmission for asthma in the all of us research program

**DOI:** 10.1186/s12967-023-04826-9

**Published:** 2024-01-04

**Authors:** Esteban Correa-Agudelo, Yadu Gautam, Angelico Mendy, Tesfaye B. Mersha

**Affiliations:** 1 Division of Asthma Research, Department of Pediatrics, Cincinnati Children’s Hospital Medical Center, University of Cincinnati College of Medicine, 3333 Burnet Ave, Cincinnati, OH 45229 USA; 2 Division of Epidemiology, Department of Environmental and Public Health Sciences, University of Cincinnati College of Medicine, Cincinnati, OH USA

**Keywords:** Length of stay, Readmission, Asthma, Comorbidities, Bayesian mixed-effects, Causal inference, Racial disparities

## Abstract

**Background:**

This study addresses the limited research on racial disparities in asthma hospitalization outcomes, specifically length of stay (LOS) and readmission, across the U.S.

**Methods:**

We analyzed in-patient and emergency department visits from the All of Us Research Program, identifying various risk factors (demographic, comorbid, temporal, and place-based) associated with asthma LOS and 30-day readmission using Bayesian mixed-effects models.

**Results:**

Of 17,233 patients (48.0% White, 30.7% Black, 19.7% Hispanic/Latino, 1.3% Asian, and 0.3% Middle Eastern and North African) with 82,188 asthma visits, Black participants had 20% shorter LOS and 12% higher odds of readmission, compared to White participants in multivariate analyses. Public-insured patients had 14% longer LOS and 39% higher readmission odds than commercially insured patients. Weekend admissions resulted in a 12% shorter LOS but 10% higher readmission odds. Asthmatics with chronic diseases had a longer LOS (range: 6–39%) and higher readmission odds (range: 9–32%) except for those with allergic rhinitis, who had a 23% shorter LOS.

**Conclusions:**

A comprehensive understanding of the factors influencing asthma hospitalization, in conjunction with diverse datasets and clinical-community partnerships, can help physicians and policymakers to systematically address racial disparities, healthcare utilization and equitable outcomes in asthma care.

**Supplementary Information:**

The online version contains supplementary material available at 10.1186/s12967-023-04826-9.

## Background

In U.S., asthma is a significant public health problem affecting 25 million people [1]. According to the Centers for Disease Control and Prevention (CDC), asthma registers more than 14 million doctors’ office visits, two million emergency department (ED) visits, half a million hospitalizations and about 3500 deaths, annually [2]. These translate to an annual economic burden that exceeds $82 billion in medical expenses, missed work and school days, and deaths [3]. Despite advances in clinical practice [4], the median length of stay (LOS) for asthma hospitalizations has remained unchanged for the last 20 years. Clinical practice, clinical history, and socio-environmental risk factors (e.g. socio-economic status, insurance, education, traffic-related air pollution, mold, pollen, and cigarette smoke) contribute to racial disparities in asthma hospitalization [4, 5]. For example, African Americans (AAs) remain four times more likely to be hospitalized and seven times more likely to die from asthma compared to European Americans (EAs) [6]. In addition, asthma is often accompanied by allergic comorbidities, such as allergic rhinitis, atopic dermatitis, and food allergy in children, as well as non-allergic disorders such as obesity, gastro-esophageal reflux, and mental health disorders [7]. How these intrinsic and extrinsic risk factors differentially impact asthma LOS and readmission among racial groups remain poorly understood.

Previous studies have investigated risk factors and racial differences associated with LOS and readmission in asthma. Chen et al. conducted a systematic review of single-center studies examining asthma LOS and clinical pathways [8]. In multi-site studies, the Hospital Episode Statistics (HES) cohort was one of the first to investigate risk factors associated to asthma LOS in London hospitals [9]. Similarly, Shanley et al. identified factors associated with LOS for pediatric asthma hospital admissions in the Pediatric Health Information System (PHIS) database [10]. More recently, Kaiser et al., reported the difficult of asthma LOS improvement using clinical pathways in a diverse, national group of hospitals in the U.S [11]. Despite the well-documented evidence of race differences and risk factors in asthma hospitalization, current studies have been primarily focused on European Americans in the U.S. Additionally, current studies are limited to small sample size and fewer risk factors due to lack of standardized data across different institutions.

These limitations may affect the unique position of physicians and policymakers on understanding the factors influencing asthma LOS and readmission to improve healthcare cost and asthma care. Therefore, we sought to characterize clinical and socioeconomic factors associated to asthma LOS in the U.S. adults using the All of Us Research Program data (hereafter termed as All of Us). All of Us, funded by the National Institutes of Health (NIH), is a cohort study aiming to enroll one million U.S. adults, particularly from underrepresented groups. Currently, the program has enrolled 413,457 participants from different races, ethnicities, age groups, and regions of the country, and collects data through survey questionnaires, electronic health records, physical measurements, linked exposures, and biospecimens (https://allofus.nih.gov/). By using a large and diverse database, this study aims to aid physicians and policymakers to better understand race differences in asthma LOS and readmission, to inform future policy initiatives aimed at improving healthcare costs and asthma care at the national level [12].

## Methods

### Study design and population

**Fig. 1:**
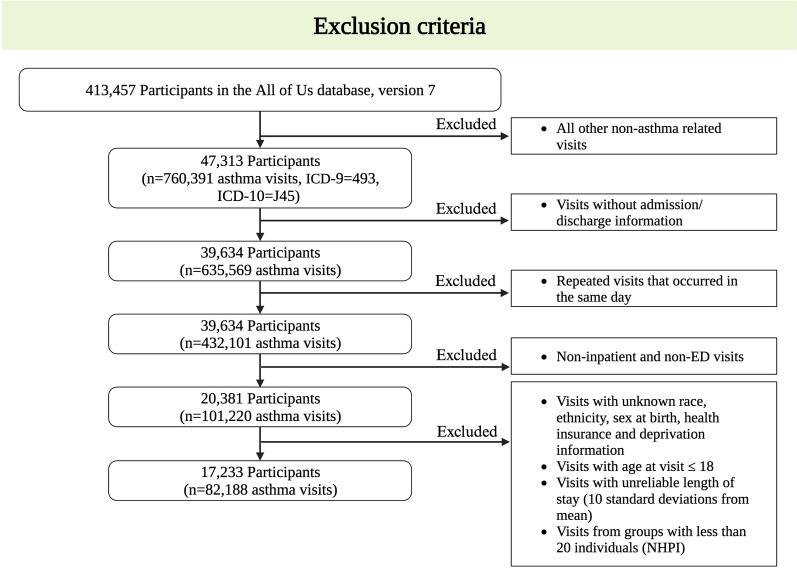
Flowchart of in-patient and asthma ED visit inclusion and exclusion criteria. The final study population includes a total of 17,233 children with 82,188 asthma ED visit

Participants with asthma from over 340 recruitment sites nationwide were identified in the All of Us database version 7, using the International Classification of Diseases, 9th and 10th revisions codes for asthma diagnoses (ICD-9-496 and ICD-10-J45). For the processing of hospital admissions, if two recorded asthma visits happened on the same day, they were considered to have the same first visit date. We only included inpatient and ED visits, and excluded those visits with missing admission discharge/phenotype information and unreliable length of stay in hospital (Ten standard deviations away from the mean). Figure 1 shows the workflow for the exclusion criteria. Additional information about the Strengthening the Reporting of Observational Studies in Epidemiology (STROBE) checklist is provided in the Additional file 1 (Table S1).

### Asthma length of stay and readmission

Length of stay per admission (LOS) is a surrogate measure of hospital utilization and refers to the number of days spent in the hospital (i.e., the difference in days between the first date of admission and the last date of discharge) [12, 13]. For our dataset, asthma LOS per admission can be zero for discharge in the same day, or larger if the patient required additional time to discharge. Furthermore, we considered the readmission of asthma patients within 30 days, inclusive of the length of stay (LOS), as a secondary outcome measure. This measure is defined as the difference in start dates between two consecutive events for a single patient, provided there is no overlap between the events [14].

### Covariates

The following individual-level risk factors (demographics, comorbidities, temporal and place-based risk) for asthma were chosen based on their association with the condition in prior research and their availability in the EHR database [10, 15–18].

### Demographic

The EHR data collected encompassed demographic variables such as sex at birth (classified as female or male), age at visit, and self-reported race/ethnicity (classified as Black, White, Hispanic/Latino [HL], or Middle Eastern and North African [MENA]. Survey questionnaires were used to infer individual-level health insurance information (classified as private or public).

### Comorbidities

The current list of comorbidities was selected according to an evidence synthesis process of relevant references [10, 16, 17] Using the appropriate ICD-9 and ICD-10 codes, our list of clinical diagnoses included atopic dermatitis (AD), allergic rhinitis (AR), cancer, coronary heart disease (CHD), chronic kidney disease (CKD), chronic obstructive pulmonary disease (COPD), depression, diabetes mellitus, eosinophilic esophagitis (EoE), food allergy (FA), gastro-esophageal reflux disease (GERD), hypertension (HTA), obesity, psoriasis, and sleep apnea. We developed a patient-level dummy variable to track clinical history of comorbidities prior to the subject’s visit (“Yes” if the patient has been previously diagnosed with the comorbid-specific disease and “No” otherwise). For instance, in the asthma encounter, if a specific patient had a previous diagnosis of any comorbidity, the comorbid-specific dummy variable for their asthma encounter was set to “Yes” accordingly. Full list of ICD-9 and ICD-10 codes is included in the Additional file 1 (Table S2).

### Temporal and place based

Temporal data on date of admission refers to weekday (Monday to Friday) or weekend (Saturday and Sunday). We categorized visit data by meteorological seasons: winter (December, January, and February), spring (March, April, and May), summer (June, July, and August), and fall (September, October, and November). All of Us database includes area-level estimation of material deprivation linked to the latest known patient zip-code. Details about this composite socioeconomic index can be found elsewhere [19].

### Statistical analysis

**Fig. 2:**
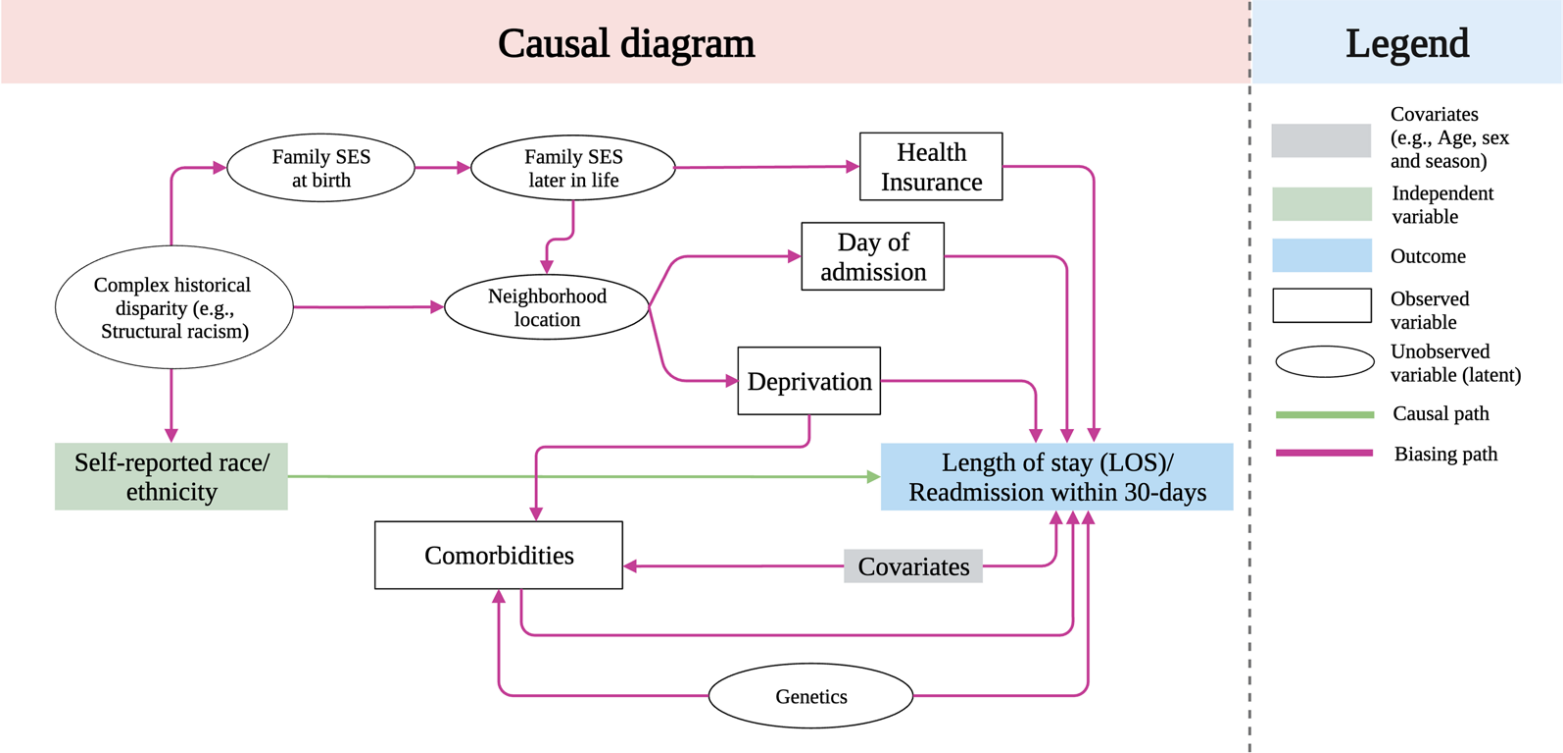
Directed acyclic graph (DAG) for the independent variable (race, clinical history of comorbidities, temporal, place-based deprivation) and the dependent variable (Length of stay [LOS], and readmission). Causal pathways, covariates, confounders, and unobserved (latent) variables are presented

Descriptive statistics were calculated for continuous (means with SD) and categorical (frequencies with percentages) variables and stratified by race/ethnicity. Associations between race/ethnicity and each variable were tested using chi-square (${\mathrm{\rm X}}^{2}$) or $t$ tests, as appropriate. To assess the association of risk factors with asthma LOS and readmission, we constructed a directed acyclic graph (DAG) with backdoor criterion to aid covariate adjustment following causal inference principles and our previous work (Fig. 2) [20–22]. We employed Bayesian mixed-effects negative binomial regression models to estimate the LOS in days. Additionally, we utilized logistic regression models to predict readmissions within a 30-day period. Both of these models were used in their univariate and multivariate forms and continuous variables (age and deprivation) were centered for numerical stability. Both, univariate and multivariate models account for subject-specific and multi-site variance including random intercepts for patients with recurrent asthma-related visits, and for EHR sites. Multivariate models included only significant risk factors, and the interaction effect between age and sex because of their significant association with asthma and included comorbidities [15]. Finally, we report Bayesian credible intervals (CI) using a 95% probability. The Methods section in this article’s Online Repository provides additional details about model formulation, univariate expected LOS and readmission, models formulation, posterior predictive distribution diagnostics, and priors used (Additional file 1: Figures S1–S2). Data extraction was performed on Python using bigquery, analyses were conducted using the R environment, ggplot for graphics, rstanarm for multivariate modelling [23].

### Model assessment

We compared Bayesian multivariate expected length of stay (LOS) and readmission models (null and adjusted set of covariates) using the expected log predictive density (ELPD) [24]. ELPD quantifies the theoretical expected log pointwise predictive density for new observations, where a higher ELPD score indicates better model fit. Because our models account for recurrent asthma-related visits, we adopted a leave-one-group-out (LOGO) scheme in the cross-validation computation (one patient, multiple asthma events) (Additional file 1).

## Results

### Race differences in asthma length of stay and readmission

Table 1 presents descriptive statistics of the All of Us’ study sample. After exclusion criteria, the cohort was composed of 17,233 individuals (48.0% White, 30.7% Black, 19.7% HL, 1.3% Asian, 0.3% MENA). Included patients contributed with 82,188 total asthma-related visits (40.9% Black, 37.4% White, 20.7% HL, 0.7% Asian, and 0.3% MENA). Lower than average asthma LOS per admission were observed in Black (1.8 days) and HL (1.8 days) (P < 0.001). Likewise, higher readmission proportions per encounter were observed in both, Black (23.6%) and HL (20.8%) groups (P < 0.001).

**Table 1:** In-patient and ED asthma visits by self-reported race including demographic, clinical history of comorbidities, temporal and place-based risk factors

Characteristics	Overall	Asian	Black	HL	MENA	White	P-Value
Patients (%)	17,233	216 (1.3)	5293 (30.7)	3394 (19.7)	56 (0.3)	8274 (48.0)	
Visits (%)	82,188	535 (0.7)	33,620 (40.9)	17,034 (20.7)	242 (0.3)	30,757 (37.4)	
LOS per admission (mean (SD))	2.0 (4.09)	2.5 (4.8)	1.8 (4.0)	1.8 (3.8)	2.1 (3.4)	2.2 (4.3)	< 0.001
Readmission within 30 days (%)							
No	64,326 (78.3)	455 (85.0)	25,694 (76.4)	13,483 (79.2)	202 (83.5)	24,492 (79.6)	< 0.001
Yes	17,862 (21.7)	80 (15.0)	7926 (23.6)	3551 (20.8)	40 (16.5)	6265 (20.4)	
Age at visit (mean (SD))	47.8 (15.1)	47.6 (17.1)	46.6 (13.4)	45.7 (15.5)	48.9 (17.2)	50.2 (16.2)	< 0.001
Sex at birth (%)							
Female	63,419 (77.2)	422 (78.9)	25,292 (75.2)	14,010 (82.2)	122 (50.4)	23,573 (76.6)	< 0.001
Male	18,769 (22.8)	113 (21.1)	8328 (24.8)	3024 (17.8)	120 (49.6)	7184 (23.4)	
Health insurance							
Private	17,319 (21.1)	204 (38.1)	5161 (15.4)	3065 (18.0)	38 (15.7)	8851 (28.8)	< 0.001
Public	64,869 (78.9)	331 (61.9)	28,459 (84.6)	13,969 (82.0)	204 (84.3)	21,906 (71.2)	
Temporal and place-based							
Season (%)							
Summer	20,276 (24.7)	125 (23.4)	8376 (24.9)	4145 (24.3)	45 (18.6)	7585 (24.7)	0.16
Fall	20,336 (24.7)	134 (25.0)	8230 (24.5)	4201 (24.7)	68 (28.1)	7703 (25.0)	
Winter	20,410 (24.8)	123 (23.0)	8401 (25.0)	4280 (25.1)	72 (29.8)	7534 (24.5)	
Spring	21,166 (25.8)	153 (28.6)	8613 (25.6)	4408 (25.9)	57 (23.6)	7935 (25.8)	
Day of admission (%)							
Weekday	63,661 (77.5)	427 (79.8)	25,659 (76.3)	13,430 (78.8)	181 (74.8)	23,964 (77.9)	< 0.001
Weekend	18,527 (22.5)	108 (20.2)	7961 (23.7)	3604 (21.2)	61 (25.2)	6793 (22.1)	
Deprivation (mean (SD))	0.33 (0.07)	0.31 (0.06)	0.35 (0.07)	0.36 (0.07)	0.35 (0.06)	0.30 (0.05)	< 0.001
Comorbidities							
AD (%)							
No	78,068 (95.0)	507 (94.8)	32,004 (95.2)	16,095 (94.5)	214 (88.4)	29,248 (95.1)	< 0.001
Yes	4120 (5.0)	28 (5.2)	1616 (4.8)	939 (5.5)	28 (11.6)	1509 (4.9)	
AR (%)							
No	57,085 (69.5)	435 (81.3)	25,234 (75.1)	11,655 (68.4)	178 (73.6)	19,583 (63.7)	< 0.001
Yes	25,103 (30.5)	100 (18.7)	8386 (24.9)	5379 (31.6)	64 (26.4)	11,174 (36.3)	
Cancer (%)							
No	44,989 (54.7)	311 (58.1)	20,662 (61.5)	9556 (56.1)	143 (59.1)	14,317 (46.5)	< 0.001
Yes	37,199 (45.3)	224 (41.9)	12,958 (38.5)	7478 (43.9)	99 (40.9)	16,440 (53.5)	
CHD (%)							
No	59,527 (72.4)	435 (81.3)	24,089 (71.7)	12,797 (75.1)	155 (64.0)	22,051 (71.7)	< 0.001
Yes	22,661 (27.6)	100 (18.7)	9531 (28.3)	4237 (24.9)	87 (36.0)	8706 (28.3)	
CKD (%)							
No	71,684 (87.2)	451 (84.3)	28,729 (85.5)	14,973 (87.9)	223 (92.1)	27,308 (88.8)	< 0.001
Yes	10,504 (12.8)	84 (15.7)	4891 (14.5)	2061 (12.1)	19 (7.9)	3449 (11.2)	
COPD (%)							
No	56,674 (69.0)	441 (82.4)	22,022 (65.5)	11,973 (70.3)	183 (75.6)	22,055 (71.7)	< 0.001
Yes	25,514 (31.0)	94 (17.6)	11,598 (34.5)	5061 (29.7)	59 (24.4)	8702 (28.3)	
Depression (%)							
No	35,495 (43.2)	391 (73.1)	16,410 (48.8)	7886 (46.3)	143 (59.1)	10,665 (34.7)	< 0.001
Yes	46,693 (56.8)	144 (26.9)	17,210 (51.2)	9148 (53.7)	99 (40.9)	20,092 (65.3)	
Diabetes (%)							
No	52,065 (63.3)	400 (74.8)	20,080 (59.7)	10,747 (63.1)	166 (68.6)	20,672 (67.2)	< 0.001
Yes	30,123 (36.7)	135 (25.2)	13,540 (40.3)	6287 (36.9)	76 (31.4)	10,085 (32.8)	
EoE (%)							
No	81,782 (99.5)	535 (100.0)	33,495 (99.6)	16,989 (99.7)	242 (100.0)	30,521 (99.2)	< 0.001
Yes	406 (0.5)	0 (0.0)	125 (0.4)	45 (0.3)	0 (0.0)	236 (0.8)	
FA (%)							
No	79,717 (97.0)	524 (97.9)	32,866 (97.8)	16,521 (97.0)	239 (98.8)	29,567 (96.1)	< 0.001
Yes	2471 (3.0)	11 (2.1)	754 (2.2)	513 (3.0)	3 (1.2)	1190 (3.9)	
GERD (%)							
No	37,165 (45.2)	343 (64.1)	16,157 (48.1)	9119 (53.5)	125 (51.7)	11,421 (37.1)	< 0.001
Yes	45,023 (54.8)	192 (35.9)	17,463 (51.9)	7915 (46.5)	117 (48.3)	19,336 (62.9)	
HTA (%)							
No	31,021 (37.7)	262 (49.0)	11,022 (32.8)	7557 (44.4)	121 (50.0)	12,059 (39.2)	< 0.001
Yes	51,167 (62.3)	273 (51.0)	22,598 (67.2)	9477 (55.6)	121 (50.0)	18,698 (60.8)	
Obesity (%)							
No	45,006 (54.8)	478 (89.3)	17,686 (52.6)	9931 (58.3)	180 (74.4)	16,731 (54.4)	< 0.001
Yes	37,182 (45.2)	57 (10.7)	15,934 (47.4)	7103 (41.7)	62 (25.6)	14,026 (45.6)	
Psoriasis (%)							
No	79,314 (96.5)	523 (97.8)	33,207 (98.8)	16,488 (96.8)	231 (95.5)	28,865 (93.8)	< 0.001
Yes	2874 (3.5)	12 (2.2)	413 (1.2)	546 (3.2)	11 (4.5)	1892 (6.2)	
Sleep apnea (%)							
No	59,240 (72.1)	444 (83.0)	24,583 (73.1)	13,527 (79.4)	191 (78.9)	20,495 (66.6)	< 0.001
Yes	22,948 (27.9)	91 (17.0)	9037 (26.9)	3507 (20.6)	51 (21.1)	10,262 (33.4)	

*LOS* Length of stay, *SD* Standard deviation, *AD* Atopic dermatitis, *AR* Allergic rhinitis, *CHD* Coronary heart disease, *CKD* Chronic kidney disease, *COPD* Chronic obstructive pulmonary disease, *EoE* Eosinophilic esophagitis, *FA* Food allergy, *GERD* Gastro-esophageal reflux disease, *HL* Hispanic/Latino, *HTA* Hypertension, *MENA* Middle Eastern and North Africa

Compared to the average, HL individuals registered the youngest average age at asthma encounter (45.7 years old), followed by Black (46.6 years old), Asian (47.6 years old), MENA (48.9 years old), and White (50.2 years old), (P < 0.001). For day of admission, MENA registered the highest admission proportion during the weekend (25.2%), followed by black (23.7%), white (22.1%), HL (21.2%), and Asian (20.2%) (P < 0.001). There were not differences in season of admission (P = 0.16). Compared to registered deprivation in White (0.30), deprivation in asthma visits was slightly higher in all the other groups (0.31 for Asian; 0.35 for Black, and MENA; and 0.36 for HL), (P < 0.001).

Among asthma visits with previous comorbidities, White participants had the highest proportions with previous AR (36.3%), cancer (53.5%), depression (65.3%), EoE (0.8%), FA (3.9%), GERD (62.9%), psoriasis (6.2%), sleep apnea (33.4%) diagnoses. Black individuals had the highest proportion of asthma visits with previous COPD (34.5%), diabetes (40.3%), HTA (67.2%), and obesity (47.4%) diagnosis; MENA individuals registered the highest proportion of asthma visits with previous AD (11.6%) and CHD (36.0%) diagnoses; and Asian individuals had the highest proportions with previous CKD (15.7%) diagnosis (all P < 0.001).

### Risk factors for asthma length of stay (LOS)

Table 2 summarizes the results for the association between asthma LOS and the assessed independent variables. Season of admission, deprivation, previous AD, EoE, FA, and Psoriasis were not associated to asthma LOS in the univariate analysis (Table S3). After adjustment, older patients were more likely to have longer LOS (Females, Expected Lengh of Stay [LOS] = 1.06, 95% credible interval [CI] = 1.03 to 1.08; Males, LOS = 1.08, CI = 1.04 to 1.12). Black and HL participants had 20% (LOS = 0.80, CI = 0.75 to 0.84) and 27% (LOS = 0.73, CI = 0.69 to 0.78) less days in hospital stay per admission compared to White individuals. Likewise, public-insured individuals had 14% additional days in hospital stay (LOS = 1.14, CI 1.09 to 1.20) compared to commercially-insured counterparts. Being admitted during the weekend had 12% less days in hospital stay (LOS = 0.88; CI = 0.85 to 0.90) compared to a weekday admission. Examining previously diagnosed comorbidities, CHD, CKD, COPD, depression, obesity, and sleep apnea had increased asthma LOS in hospital (range: 6% to 39% additional days) except for those with AR, who had a 23% shorter LOS (LOS = 0.77; CI = 0.74 to 0.79) per asthma admission.

**Table 2:** Bayesian multivariate expected length of stay (LOS) and readmission within 30 days showing median and 95% credible intervals for associations between asthma hospitalizations and significant risk factors (demographics, individual- comorbidities, temporal and place-based)

Characteristic	Expected LOS, 95% CI	Readmission OR, 95% CI
Age at visit by Sex		
Female	**1.06 (1.03 to 1.08)**	**0.70 (0.67 to 0.73)**
Male	**1.08 (1.04 to 1.12)**	**0.75 (0.70 to 0.80)**
Race alone		
White EA	1 (Ref)	1 (Ref)
Asian	1.0 (0.83 to 1.22)	1.0 (0.7 to 1.4)
Black AA	**0.80 (0.75 to 0.84)**	**1.12 (1.03 to 1.21)**
HL	**0.73 (0.69 to 0.78)**	1.02 (0.92 to 1.13)
MENA	0.85 (0.61 to 1.15)	1.13 (0.68 to 1.82)
Insurance		
Private	1 (Ref)	1 (Ref)
Public	**1.14 (1.09 to 1.20)**	**1.39 (1.29 to 1.50)**
Day of admission		
Weekday	1 (Ref)	1 (Ref)
Weekend	**0.88 (0.85 to 0.90)**	**1.10 (1.05 to 1.15)**
Deprivation	–	1.0 (0.96 to 1.05)
Comorbidities		
AR		
No	1 (Ref)	1 (Ref)
Yes	**0.77 (0.74 to 0.79)**	0.96 (0.9 to 1.02)
Cancer		
No	1 (Ref)	1 (Ref)
Yes	1.04 (1.0 to 1.07)	–
CHD		
No	1 (Ref)	1 (Ref)
Yes	**1.20 (1.16 to 1.25)**	**1.14 (1.07 to 1.22)**
CKD		
No	1 (Ref)	1 (Ref)
Yes	**1.39 (1.32 to 1.45)**	**1.09 (1.01 to 1.18)**
COPD		
No	1 (Ref)	1 (Ref)
Yes	**1.07 (1.03 to 1.10)**	**1.32 (1.24 to 1.40)**
Depression		
No	1 (Ref)	1 (Ref)
Yes	**1.06 (1.02 to 1.09)**	**1.32 (1.25 to 1.40)**
Diabetes		
No	1 (Ref)	1 (Ref)
Yes	1.04 (1.0 to 1.08)	1.05 (0.99 to 1.12)
FA		
No	1 (Ref)	1 (Ref)
Yes	–	1.08 (0.93 to 1.28)
GERD		
No	1 (Ref)	1 (Ref)
Yes	1.03 (1.0 to 1.07)	**1.21 (1.14 to 1.28)**
HTA		
No	1 (Ref)	1 (Ref)
Yes	**1.07 (1.03 to 1.11)**	**1.28 (1.21 to 1.37)**
Obesity		
No	1 (Ref)	1 (Ref)
Yes	**1.09 (1.05 to 1.13)**	1.0 (0.94 to 1.06)
Psoriasis		
No	1 (Ref)	1 (Ref)
Yes	–	1.16 (0.99 to 1.33)
Sleep apnea		
No	1 (Ref)	1 (Ref)
Yes	**1.06 (1.02 to 1.10)**	1.01 (0.95 to 1.07)

Bold denotes significant posteriors

*LOS* Length of stay, *OR* Odds ratio, *CI* Credible interval, *AR* Allergic rhinitis, *CHD* Coronary heart disease, *CKD* Chronic kidney disease, *COPD* Chronic obstructive pulmonary disease, *FA* Food allergy, *GERD* Gastro-esophageal reflux disease, *HL* Hispanic/Latino, *HTA* Hypertension, *MENA* Middle Eastern and North Africa

### Risk factors for asthma readmission

Table 2 summarizes the results for the impact of asthma LOS in readmission and the assessed risk factors. LOS, Season of admission, previous AD, cancer, and EoE were not associated to asthma readmission in the univariate analysis (Table S3). After adjustment, younger patients had higher odds of readmission (Females, Odds ratio [OR] = 0.70, 95% credible interval [CI] = 0.67 to 0.73; Males, OR = 0.75, CI = 0.70 to 0.80). Black individuals had 12% higher odds of asthma readmission (OR = 1.12, CI = 1.03 to 1.21) compared to White individuals. Likewise, public-insured individuals had 39% higher odds of asthma readmission (OR = 1.39, CI = 1.29 to 1.50) compared to commercially-insured counterparts. Being admitted during the weekend had 10% higher odds of readmission (OR = 1.10, CI = 1.05 to 1.15) compared to a weekday admission. Examining previously diagnosed comorbidities in readmission, CHD, CKD, COPD, depression, GERD, and HTA, were associated with higher odds of asthma readmission (range: 9–32%).

### Validation

Table S4 shows validation performance in expected length of stay (LOS) and readmission. Adjusted models had better ELPD in both outcomes (LOS and readmission) compared to the null (ELPD diff > 4).

## Discussion

Here we report differences in demographic, comorbidities, and temporal and place-based risk factors for asthma LOS and readmission in a large and diverse All of Us dataset. Compared to White, Black and Hispanic/Latino (HL) experienced shorter than average asthma LOS, and higher proportions of readmission. Our multivariate analyses suggest that race differences in asthma LOS and readmission were mainly driven by health insurance, and day of admission. Patients with clinical history of several chronic diseases were associated with longer LOS and increased odds of readmission, whereas patients with previous AR were associated with shorter LOS.

In our multivariate analyses, we found differences in LOS and probability of readmission among different racial groups. Specifically, Black and HL adults had shorter LOS, but only Black individuals showed increased odds of asthma readmission compared to the White individuals. This aligns with previous studies from our group and others, which have frequently reported worse asthma outcomes among minority populations [21, 22, 25]. We hypothesize that certain factors indirectly related to race, such as health insurance status, and day of admission, could explain the observed negative correlation between shorter LOS due to an early discharge and the increased odds of readmission among black patients. Such factors have been linked to decreased quality of care in other studies. For example, the so-called “weekend effect”—the phenomenon of patients admitted over the weekend being discharged earlier—influenced by various patient and health service-related factors (e.g., quality of care, medication errors, bed occupancy rates, staff levels, disease management education) could result in posterior readmissions [10, 26, 27]. Unfortunately, these factors are more prevalent among low-income groups who often seek care on weekends due to limited options (e.g., accessibility barriers). Links between age and related asthma outcomes are consistent with the literature including our group [15, 22]. Such links also extend to other comorbidities evaluated in our manuscript [10, 16, 17]. Further research is needed to explore these relationships specifically in asthma, and develop strategies (e.g., telemedicine, internal processes and EHR, attending schedules and nighttime in clinical settings) [28, 29] to improve asthma outcomes among these vulnerable populations.

Our findings revealed that having a clinical history of comorbidities is associated with asthma LOS and readmission differences. We observed a group consisting of chronic diseases (CHD, CKD, COPD, depression, GERD, HTA, obesity, and sleep apnea) that may prolong asthma LOS or increase odds of asthma readmission, and a second group consisting of allergic diseases such as AR that may shorten it. The former group has been well-documented with supported evidence as major comorbid diseases with asthma. For example, Black and Latino asthmatics are disproportionally affected by asthma related comorbidities including obesity or chronic diseases [17, 30]. Our study could help physicians to develop targeted interventions such as a clinical risk profile in daily practice leading to earlier diagnosis and treatment of comorbidities with subsequent improvement in outcome and reduce the asthma burden, especially in minorities. The latter group could be explained by the fact that asthma events in individuals with a clinical history of Type 2 immune disorders may be more frequent as previously reported by others authors, but not necessarily more severe [31–33]. Previous research has shown that treatment for AR (e.g., inhaled/oral steroids or cromolyn) can reduce the risk of hospitalization and ER visits for asthma patients [34]. We hypothesize asthma patients with a co-occurrence of AR are more frequently admitted in hospital for asthma but may recover more quickly once admitted due to their beneficial treatment impacts, exhibiting a shorter hospital stay. However, effects from disease-specific comorbidities on asthma are remain poorly understood and require further investigation to achieve a reduction in asthma healthcare utilization.

There are a number of limitations associated with this study. First, although this study was conducted in a large and diverse admission/discharge standardized dataset across more than 340 locations in the U.S., the findings may not be generalizable to non-All of Us member institutions. Second, race/ethnicity was determined based on self-reported information collected and stored in the electronic health record (EHR). We recognize that self-reported race is a social construct and does not reflect genetic ancestry. As such, it should be considered solely as a sociopolitical construct, rather than a marker of biological differences [35]. We also recognize that the sample size for Asian and Middle Eastern and North African are relatively small. Future research could include more samples from these population, which is becoming increasingly available in the All of Us program [36]. Third, we had limited individual-level data available within the EHR that may affect an accurate estimation of LOS. Future research might include potential clinical pathway information about the hospital stay (e.g., physiologic readiness for discharge [PRD], bronchodilator/corticosteroid usage, and oxygen saturation) that were not available in our study [37, 38]. Fourth, due to the lack of precise patient-level environmental data, our estimate of place-based deprivation, based only on the last known area-level location. This may not accurately capture patient-level socioeconomic context specially those who change their physical address. Also, using place-based deprivation as a proxy of patient-level socioeconomic context might lead to bias (ecological fallacy) [39]. Finally, this study relied on routinely collected ICD-9/ICD-10 billing codes associated to asthma. Other unobserved clinical and health-related factors that occur during the admission discharge window could influence the variation in length of stay.

## Conclusions

To our knowledge, this is the first study to characterize demographic, clinical history of comorbidities, temporal and socioeconomic risk factors associated to asthma LOS and asthma readmission in a large and diverse cohort as All of Us. We reported more than 82,000 recurrent in-patient and ED hospital admissions from about 17,000 U.S. adults including underrepresented groups to examine epidemiological differences in asthma LOS and asthma readmission from a broad range of variables with an explicit causal thinking. Some public health policies to address racial disparities could be derived from this study. First, promote the “meaningful use” of the electronic health record in the care of asthmatic patients. For example, exploring integration and the optimal use of the electronic health record to tailor clinical pathways and health interventions could help improve access and asthma care for groups at risk [40]. Second, clinical-community partnerships can collaborate to systematically address modifiable place-based risk factors improving health and well-being. Strategies to link clinical partners and communities aimed to reduce health disparities have been successfully documented and can be adapted to adult asthma population (41). We identified how asthma LOS and asthma readmission differs by race, health insurance, clinical history of comorbidities, and day of admission. The identification of these factors in a multi-site and diverse cohort could provide potential areas of future research aimed to reduce healthcare utilization and achieve equitable outcomes in asthma-related morbidity across the U.S.

### Supplementary Information

**Additional file 1: Table S1.** STROBE Statement—checklist of items that should be included in reports of observational studies. **Table S2.** ICD-9/ICD-10 billing codes used to search asthma and comorbidities diagnoses.** Table S3.** Univariate expected length of stay (LOS) and readmission within 30-days showing median and 95% credible intervals for associations between asthma hospitalizations and significant risk factors (demographics, individual- comorbidities, temporal and place-based).** Table S4.** Validation performance summary in expected length of stay (LOS) and readmission. We compare ELPD using a Leave-one-group-out (LOGO) scheme in two statistical models (null and adjusted set of covariates).** Figure S1.** Trace rank plots for chains distribution in the multivariate asthma LOS and readmission models. Intertwined chain lines for intercept parameter mean MCMC chains are exploring parameter space efficiently. **A** Asthma LOS; **B** Asthma readmission within 30-days.** Figure S2.** Pareto smoothed importance sampling (PSIS) for multivariate asthma LOS and readmission models: **A** Asthma LOS model exhibited about of 21.1% of subject-specific observations over-optimistic inference; **B** Asthma readmission within 30-days showed about of 6.1% of subject-specific observations over-optimistic inference.

## Data Availability

All methods were carried out in accordance with relevant All of Us guidelines and regulations. R/Python code for this article has been annotated and deposited as open-source code in GitHub at https://github.com/maurosc3ner/los_readmission_allofus_2023.

## Abbreviations

AD: Atopic dermatitis
AR: Allergic rhinitis
CHD: Coronary heart disease
CI: Credible Interval
CDC: Centers for Disease Control and Prevention
CKD: Chronic kidney disease
COPD: Chronic obstructive pulmonary disease
DAG: Directed acyclic graph
DoA: Day of admission
EHR: Electronic health records
EoE: Eosinophilic Esophagitis
FA: Food allergy
GERD: Gastro-esophageal reflux disease
ICD-9: International Classification of Diseases, 9th Revision
ICD-10: International Classification of Diseases, 10th Revision
LOS: Length of stay
MENA: Middle Eastern and North African
NIH: National Institutes of Health
PHIS: Pediatric Health Information System

## References

[1] Akinbami LJ, Bailey C, Zahran HS, King M, Johnson CA, Liu X (2012). Trends in asthma prevalence, health care use, and mortality in the United States, 2001–2010. Centers Dis Control Prev.

[2] CDC (2019). Most Recent National Asthma Data: Centers for Disease Control and Prevention.

[3] Nurmagambetov T, Kuwahara R, Garbe P (2018). The economic burden of asthma in the United States, 2008–2013. Ann Am Thorac Soc.

[4] NAEPP (2007). Guidelines for the Diagnosis and Management of Asthma (EPR-3).

[5] Macy ML, Stanley RM, Lozon MM, Sasson C, Gebremariam A, Davis MM (2009). Trends in high-turnover stays among children hospitalized in the United States, 1993–2003. Pediatrics.

[6] Akinbami LJ, Moorman JE, Simon AE, Schoendorf KC (2014). Trends in racial disparities for asthma outcomes among children 0 to 17 years, 2001–2010. J Allergy Clin Immunol.

[7] Holgate ST, Wenzel S, Postma DS, Weiss ST, Renz H, Sly PD (2015). Asthma. Nat Rev Dis Primers.

[8] Chen K-H, Chen C, Liu H-E, Tzeng P-C, Glasziou PP. Effectiveness of paediatric asthma clinical pathways: a narrative systematic review. Journal of Asthma. 2014. 51(5):480–92. 10.3109/02770903.2014.887728 24471514

[9] Soyiri IN, Reidpath DD, Sarran C (2011). Asthma length of stay in hospitals in london 2001–2006: demographic, diagnostic and temporal factors. PLoS ONE.

[10] Shanley LA, Lin H, Flores G (2015). Factors associated with length of stay for pediatric asthma hospitalizations. J Asthma.

[11] Kaiser SV, Jennings B, Rodean J, Cabana MD, Garber MD, Ralston SL (2020). Pathways for Improving Inpatient Pediatric Asthma Care (PIPA): A Multicenter, National Study. Pediatrics.

[12] Silber JH, Rosenbaum PR, Even-Shoshan O, Shabbout M, Zhang X, Bradlow ET (2003). Length of stay, conditional length of stay, and prolonged stay in pediatric asthma. Health Serv Res.

[13] Faddy M, Graves N, Pettitt A (2009). Modeling length of stay in hospital and other right skewed data: comparison of phase-type, gamma and log-normal distributions. Value in Health.

[14] Bradley SV, Hall M, Rajan D, Johnston J, Ondrasek E, Chen C (2023). Sustaining long-term asthma outcomes at a community and tertiary care pediatric hospital. Hosp Pediatr.

[15] Skobeloff EM, Spivey WH, Clair SSS, Schoffstall JM (1992). The influence of age and sex on asthma admissions. JAMA.

[16] Hill TD, Graham LM, Divgi V (2011). Racial disparities in pediatric asthma: a review of the literature. Curr Allergy Asthma Rep.

[17] Mendy A, Mersha TB (2022). Comorbidities in childhood-onset and adult-onset asthma. Ann Allergy Asthma Immunol.

[18] DePriest K, Butz A (2016). Neighborhood-level factors related to asthma in children living in urban areas: an integrative literature review. J Sch Nurs.

[19] Brokamp C, Beck AF, Goyal NK, Ryan P, Greenberg JM, Hall ES (2019). Material community deprivation and hospital utilization during the first year of life: an urban population–based cohort study. Ann Epidemiol.

[20] Hernan MA, & Robins, J.M. Causal Inference: What If. Boca Raton: Chapman & Hall/CRC; 2020.

[21] Beck AF, Huang B, Auger KA, Ryan PH, Chen C, Kahn RS (2016). Explaining racial disparities in child asthma readmission using a causal inference approach. JAMA Pediatr.

[22] Correa-Agudelo E, Ding L, Beck AF, Brokamp C, Altaye M, Kahn RS (2022). Understanding racial disparities in childhood asthma using individual- and neighborhood-level risk factors. J Allergy Clinical Immunol.

[23] R Core Team (2018). R: a language and environment for statistical computing. 3.5.

[24] Vehtari A, Gelman A, Gabry J (2017). Practical Bayesian model evaluation using leave-one-out cross-validation and WAIC. Stat Comput.

[25] Biagini JM, Kroner JW, Baatyrbekkyzy A, Gonzales A, He H, Stevens M (2022). Longitudinal atopic dermatitis endotypes: An atopic march paradigm that includes Black children. J Allergy Clin Immunol.

[26] Lawson CC, Carroll K, Gonzalez R, Priolo C, Apter AJ, Rhodes KV (2014). “No other choice”: reasons for emergency department utilization among urban adults with acute asthma. Acad Emerg Med.

[27] Mahony T, Harder VS, Ang N, McCulloch CE, Shaw JS, Thombley R (2022). Weekend versus weekday asthma-related emergency department utilization. Acad Pediatr.

[28] Wong H, Wu RC, Tomlinson G, Caesar M, Abrams H, Carter MW (2009). How much do operational processes affect hospital inpatient discharge rates?. J Public Health.

[29] Blecker S, Goldfeld K, Park N, Shine D, Austrian JS, Braithwaite RS (2014). Electronic health record use, intensity of hospital care, and patient outcomes. Am J Med.

[30] Mahdavian M, Power BH, Asghari S, Pike JC (2018). Effects of comorbidities on asthma hospitalization and mortality rates: a systematic review. Can Respir J.

[31] Bergeron C, Hamid Q (2005). Relationship between asthma and rhinitis: epidemiologic, pathophysiologic, and therapeutic aspects. Allergy Asthma Clin Immunol.

[32] Pullerits TRE, Ekerljung L, Palmqvist MA, Arvidsson M, Mincheva R, Backman H, Kankaanranta H, Ilmarinen P, Rådinger M, Lundbäck B, Nwaru BI (2021). The triad of current asthma, rhinitis and eczema is uncommon among adults: Prevalence, sensitization profiles, and risk factors. Respir Med.

[33] Jaggi V, Dalal A, Ramesh B, Tikkiwal S, Chaudhry A, Kothari N (2019). Coexistence of allergic rhinitis and asthma in Indian patients: The CARAS survey. Lung India.

[34] Crystal-Peters J, Neslusan C, Crown WH, Torres A (2002). Treating allergic rhinitis in patients with comorbid asthma: The risk of asthma-related hospitalizations and emergency department visits. J Allergy Clin Immunol.

[35] Mersha TB, Abebe T (2015). Self-reported race/ethnicity in the age of genomic research: its potential impact on understanding health disparities. Hum Genomics.

[36] Zhu Z, Lee PH, Chaffin MD, Chung W, Loh P-R, Lu Q (2018). A genome-wide cross-trait analysis from UK Biobank highlights the shared genetic architecture of asthma and allergic diseases. Nat Genet.

[37] Wazeka A, Valacer DJ, Cooper M, Caplan DW, DiMaio M (2001). Impact of a pediatric asthma clinical pathway on hospital cost and length of stay*. Pediatr Pulmonol.

[38] Simmons JM, Biagini Myers JM, Martin LJ, Kercsmar CM, Schuler CL, Pilipenko VV (2018). Ohio pediatric asthma repository: opportunities to revise care practices to decrease time to physiologic readiness for discharge. Hosp Pediatr.

[39] Swift A, Liu L, Uber J (2014). MAUP sensitivity analysis of ecological bias in health studies. GeoJournal.

[40] Garg A, Toy S, Tripodis Y, Silverstein M, Freeman E (2015). Addressing social determinants of health at well child care visits: a cluster rct. Pediatrics.

[41] Henize AW, Beck AF, Klein MD, Adams M, Kahn RS (2015). A road map to address the social determinants of health through community collaboration. Pediatrics.

