# Supplementary material for: Racial differences in length of stay and readmission for asthma in the all of us research program
Source: J Transl Med. 2024 Jan 4;22:22. doi: 10.1186/s12967-023-04826-9 (PMC10768130; doi:10.1186/s12967-023-04826-9)
Supplement: Supplementary file 1 — Additional file 1: Table S1. STROBE Statement—checklist of items that should be included in reports of observational studies. Table S2. ICD-9/ICD-10 billing codes used to search asthma and comorbidities diagnoses. Table S3. Univariate expected length of stay (LOS) and readmission within 30-days showing median and 95% credible intervals for associations between asthma hospitalizations and significant risk factors (demographics, individual- comorbidities, temporal and place-based). Table S4. Validation performance summary in expected length of stay (LOS) and readmission. We compare ELPD using a Leave-one-group-out (LOGO) scheme in two statistical models (null and adjusted set of covariates). Figure S1. Trace rank plots for chains distribution in the multivariate asthma LOS and readmission models. Intertwined chain lines for intercept parameter mean MCMC chains are exploring parameter space efficiently. A Asthma LOS; B Asthma readmission within 30-days. Figure S2. Pareto smoothed importance sampling (PSIS) for multivariate asthma LOS and readmission models: A Asthma LOS model exhibited about of 21.1% of subject-specific observations over-optimistic inference; B Asthma readmission within 30-days showed about of 6.1% of subject-specific observations over-optimistic inference. [file 12967_2023_4826_MOESM1_ESM.docx]

**Online Additional file 1**

**Racial Differences in Length of Stay and Readmission for Asthma in the All of Us Research Program**

**Supplementary Methods**

**Bayesian analysis details**

Our Bayesian mixed effects models used Hamiltonian Monte Carlo No-U-Turn Sampler (NUTS) with 4 chains, and 1200 iterations (50% warmup, 50% inference). The weak informative priors for the intercept and covariates were defined as:

$${Intercept}_{i}\sim Normal\left( 0,1 \right)$$

$$\beta_{m}\sim Normal\left( 0,1 \right)$$

Regression models were developed in R, using packages *rstanarm*, and *ggplot*. We provide rank and Pareto Smoothed Importance Sampling (PSIS) plots for asthma LOS and readmission (See Figures S1-S2).

**Additional file 1: Table S1.** STROBE Statement—checklist of items that should be included in reports of observational studies

|  | **Item No** | **Recommendation** | **Page  No** |
| --- | --- | --- | --- |
| **Title and abstract** | 1 | (*a*) Indicate the study’s design with a commonly used term in the title or the abstract | Abstract, methods section |
|  |  | (*b*) Provide in the abstract an informative and balanced summary of what was done and what was found | Abstract |
| **Introduction** | | | |
| Background/rationale | 2 | Explain the scientific background and rationale for the investigation being reported | Introduction, paragraphs 1-2 |
| Objectives | 3 | State specific objectives, including any prespecified hypotheses | Introduction, paragraph 2 |
| **Methods** | | | |
| Study design | 4 | Present key elements of study design early in the paper | Material and Methods, paragraph 1 |
| Setting | 5 | Describe the setting, locations, and relevant dates, including periods of recruitment, exposure, follow-up, and data collection | Material and Methods, paragraph 1 |
| Participants | 6 | (*a*) *Cohort study*—Give the eligibility criteria, and the sources and methods of selection of participants. Describe methods of follow-up  *Case-control study*—Give the eligibility criteria, and the sources and methods of case ascertainment and control selection. Give the rationale for the choice of cases and controls  *Cross-sectional study*—Give the eligibility criteria, and the sources and methods of selection of participants | Material and Methods, paragraph 1 |
|  |  | (*b*) *Cohort study*—For matched studies, give matching criteria and number of exposed and unexposed  *Case-control study*—For matched studies, give matching criteria and the number of controls per case |  |
| Variables | 7 | Clearly define all outcomes, exposures, predictors, potential confounders, and effect modifiers. Give diagnostic criteria, if applicable | Material and Methods, paragraphs 2-6 |
| Data sources/ measurement | 8* | For each variable of interest, give sources of data and details of methods of assessment (measurement). Describe comparability of assessment methods if there is more than one group | Material and Methods, paragraphs 3-6 |
| Bias | 9 | Describe any efforts to address potential sources of bias |  |
| Study size | 10 | Explain how the study size was arrived at | Material and Methods, paragraph 1 |
| Quantitative variables | 11 | Explain how quantitative variables were handled in the analyses. If applicable, describe which groupings were chosen and why | Material and Methods, paragraphs 3-6 |
| Statistical methods | 12 | (*a*) Describe all statistical methods, including those used to control for confounding | Material and Methods, paragraph 7  Supplementary Methods |
|  |  | (*b*) Describe any methods used to examine subgroups and interactions |  |
|  |  | (*c*) Explain how missing data were addressed | Material and Methods, paragraph 1 |
|  |  | (*d*) *Cohort study*—If applicable, explain how loss to follow-up was addressed  *Case-control study*—If applicable, explain how matching of cases and controls was addressed  *Cross-sectional study*—If applicable, describe analytical methods taking account of sampling strategy |  |
|  |  | (*e*) Describe any sensitivity analyses |  |

Continued on next page

| **Results** | | | |
| --- | --- | --- | --- |
| Participants | 13* | (a) Report numbers of individuals at each stage of study—eg numbers potentially eligible, examined for eligibility, confirmed eligible, included in the study, completing follow-up, and analysed | Material and Methods, paragraph 1 |
|  |  | (b) Give reasons for non-participation at each stage | Material and Methods, paragraph 1 |
|  |  | (c) Consider use of a flow diagram | Material and Methods, paragraph 1 |
| Descriptive data | 14* | (a) Give characteristics of study participants (eg demographic, clinical, social) and information on exposures and potential confounders | Results, paragraphs 1-2 |
|  |  | (b) Indicate number of participants with missing data for each variable of interest |  |
|  |  | (c) *Cohort study*—Summarise follow-up time (eg, average and total amount) |  |
| Outcome data | 15* | *Cohort study*—Report numbers of outcome events or summary measures over time | Results, paragraph 1 |
|  |  | *Case-control study—*Report numbers in each exposure category, or summary measures of exposure |  |
|  |  | *Cross-sectional study—*Report numbers of outcome events or summary measures |  |
| Main results | 16 | (*a*) Give unadjusted estimates and, if applicable, confounder-adjusted estimates and their precision (eg, 95% confidence interval). Make clear which confounders were adjusted for and why they were included | Results, paragraphs 3-4  Supplemental methods Table S3 |
|  |  | (*b*) Report category boundaries when continuous variables were categorized |  |
|  |  | (*c*) If relevant, consider translating estimates of relative risk into absolute risk for a meaningful time period | Results, paragraphs 3-4 |
| Other analyses | 17 | Report other analyses done—eg analyses of subgroups and interactions, and sensitivity analyses |  |
| **Discussion** | | | |
| Key results | 18 | Summarise key results with reference to study objectives | Discussion, paragraph 1 |
| Limitations | 19 | Discuss limitations of the study, taking into account sources of potential bias or imprecision. Discuss both direction and magnitude of any potential bias | Discussion, paragraph 4 |
| Interpretation | 20 | Give a cautious overall interpretation of results considering objectives, limitations, multiplicity of analyses, results from similar studies, and other relevant evidence | Discussion, paragraphs 2-4 |
| Generalisability | 21 | Discuss the generalisability (external validity) of the study results | Discussion, paragraphs 4-5 |
| **Other information** | | | |
| Funding | 22 | Give the source of funding and the role of the funders for the present study and, if applicable, for the original study on which the present article is based | Front page, Acknowledgement section |

*Give information separately for cases and controls in case-control studies and, if applicable, for exposed and unexposed groups in cohort and cross-sectional studies.

**Additional file 1: Table S2.** ICD-9/ICD-10 billing codes used to search asthma and comorbidities diagnoses.

| **Diagnosis** | **ICD-9** | **ICD-10** |
| --- | --- | --- |
| Asthma | 493 | J45 |
| AD | 691 | L20 |
| AR | 477 | J30 |
| Cancer | 140 to 239 | C00-D49 |
| CHD | 410 411 412 413 414 | I20 I21 I22 I23 I24 I25 |
| CKD | 585 | N18 |
| COPD | 491.20 491.21 491.22 493.20 493.21 493.22 496 | J44 |
| Depression | 296.2 296.3 300.0 300.8 | F32 F33 F41 F43 |
| Diabetes | 250 | E10 E11 |
| EoE | 530.13 | K20.0 |
| FA | 693.1 995.6 995.7 708.0 | Z91.01 Z91.02 L50.0 |
| GERD | 530.81 | K21 |
| HTA | 401 | I10 |
| Obesity | 278.0 | E66.0 |
| Psoriasis | 696 696.1 | L40 |
| Sleep Apnea | 327.2 | G47.3 |

**Additional file 1: Table S3.** Univariate expected length of stay (LOS) and readmission within 30-days showing median and 95% credible intervals for associations between asthma hospitalizations and significant risk factors (demographics, individual- comorbidities, temporal and place-based).

| **Characteristic** | **Expected LOS 95% CI** | **OR 95% CI** |
| --- | --- | --- |
| **LOS** | — | 1.0 (1.0 to 1.0) |
| Age at visit by Sex |  |  |
| Female | **1.18 (1.16 to 1.21)** | **0.99 (0.99 to 0.99)** |
| Male | **1.24 (1.20 to 1.29)** | **0.99 (0.99 to 1.0)** |
| Race |  |  |
| White | — | — |
| Asian | 0.91 (0.76 to 1.09) | 0.93 (0.67 to 1.30) |
| Black | **0.84 (0.80 to 0.89)** | **1.31 (1.21 to 1.41)** |
| Hispanic or Latino | **0.73 (0.69 to 0.78)** | **1.22 (1.10 to 1.34)** |
| MENA | 0.81 (0.59 to 1.12) | 1.27 (0.77 to 2.10) |
| Insurance |  |  |
| Private | — | — |
| Public | **1.26 (1.20 to 1.31)** | **1.47 (1.37 to 1.58)** |
| Season (%) |  |  |
| Summer | — | — |
| Fall | 0.99 (0.96 to 1.02) | 1.05 (0.99 to 1.11) |
| Winter | 1.02 (0.99 to 1.05) | 0.98 (0.93 to 1.03) |
| Spring | 1.00 (0.97 to 1.03) | 1.02 (0.96 to 1.07) |
| Day of admission |  |  |
| Weekday | — | — |
| Weekend | **0.88 (0.85 to 0.90)** | **1.11 (1.06 to 1.16)** |
| Deprivation | 0.99 (0.96 to 1.01) | **1.05 (1.01 to 1.10)** |
| **Comorbidities** | | |
| AD |  |  |
| No | — | — |
| Yes | 0.92 (0.85 to 1.0) | 0.98 (0.86 to 1.10) |
| AR |  |  |
| No | — | — |
| Yes | **0.90 (0.87 to 0.93)** | **1.08 (1.02 to 1.14)** |
| Cancer |  |  |
| No | — | — |
| Yes | **1.18 (1.14 to 1.22)** | 0.99 (0.95 to 1.04) |
| CHD |  |  |
| No | — | — |
| Yes | **1.44 (1.39 to 1.49)** | **1.27 (1.20 to 1.34)** |
| CKD |  |  |
| No | — | — |
| Yes | **1.64 (1.57 to 1.71)** | **1.25 (1.16 to 1.34)** |
| COPD |  |  |
| No | — | — |
| Yes | **1.27 (1.23 to 1.31)** | **1.40 (1.33 to 1.48)** |
| Depression |  |  |
| No | — | — |
| Yes | **1.17 (1.13 to 1.20)** | **1.50 (1.43 to 1.58)** |
| Diabetes |  |  |
| No | — | — |
| Yes | **1.27 (1.23 to 1.31)** | **1.24 (1.18 to 1.31)** |
| EoE |  |  |
| No | — | — |
| Yes | 0.92 (0.74 to 1.15) | 1.24 (0.87 to 1.76) |
| FA |  |  |
| No | — | — |
| Yes | 1.02 (0.92 to 1.13) | **1.30 (1.11 to 1.52)** |
| GERD |  |  |
| No | — | — |
| Yes | **1.20 (1.17 to 1.24)** | **1.34 (1.28 to 1.41)** |
| HTA |  |  |
| No | — | — |
| Yes | **1.31 (1.27 to 1.35)** | **1.29 (1.23 to 1.36)** |
| Obesity |  |  |
| No | — | — |
| Yes | **1.21 (1.17 to 1.25)** | **1.23 (1.17 to 1.30)** |
| Psoriasis |  |  |
| No | — | — |
| Yes | 1.09 (1.0 to 1.19) | **1.23 (1.07 to 1.42)** |
| Sleep apnea |  |  |
| No | — | — |
| Yes | **1.26 (1.22 to 1.30)** | **1.22 (1.15 to 1.29)** |
| LOS: Length of stay; OR: Odds Ratio; CI: Credible interval; AD: Atopic dermatitis; AR: Allergic rhinitis; CHD: Coronary heart disease; CKD: Chronic kidney disease; COPD: Chronic obstructive pulmonary disease; EoE: Eosinophilic esophagitis; FA: Food allergy; GERD: Gastro-esophageal reflux disease; HTA: Hypertension; Bold denotes significant posteriors. | | |

**Additional file 1: Table S4.** Validation performance summary in expected length of stay (LOS) and readmission. We compare ELPD using a Leave-one-group-out (LOGO) scheme in two statistical models (null and adjusted set of covariates).

|  | **ELPD (LOGO)** | **ELPD difference** | **SE difference** |
| --- | --- | --- | --- |
| **Expected LOS** | | | |
| **Adjusted model** | -137,331.6 | 0.00 | 0.00 |
| **Null model** | -137,745.7 | -414.07 | 44.51 |
| **Readmission** | | | |
| **Adjusted model** | -37,128.21 | 0.00 | 0.00 |
| **Null model** | -37,410.99 | -282.77 | 32.02 |

**Additional file 1: Figure S1.** Trace rank plots for chains distribution in the multivariate asthma LOS and readmission models. Intertwined chain lines for intercept parameter mean MCMC chains are exploring parameter space efficiently. A) asthma LOS; B) Asthma readmission within 30-days.

**Additional file 1: Figure S2.** Pareto smoothed importance sampling (PSIS) for multivariate asthma LOS and readmission models: A) Asthma LOS model exhibited about of 21.1% of subject-specific observations **over-optimistic inference**; B) Asthma readmission within 30-days showed about of 6.1% of subject-specific observations **over-optimistic inference**.
